# Achievable aspiration flow rates with large balloon guide catheters during carotid artery stenting

**DOI:** 10.1186/s42155-020-00134-1

**Published:** 2020-09-10

**Authors:** Tilman Schubert, Leonardo Rivera-Rivera, Alejandro Roldan-Alzate, Daniel Consigny, Lorenz Leitner, Charles Strother, Beverly Aagaard-Kienitz

**Affiliations:** 1 Department of Radiology, University of Wisconsin-Madison, Madison, WI USA; 2 Department of Neuroradiology, Zurich University Hospital, Zurich, Switzerland; 3 Department of Medical Physics, University of Wisconsin-Madison, Madison, WI USA; 4 Department of Mechanical Engineering, University of Wisconsin-Madison, Madison, WI USA; 5 Department of Biomedical Engineering, University of Wisconsin-Madison, Madison, WI USA; 6 Department of Neuro-Urology, Balgrist University Hospital, Zurich, Switzerland; 7 Department of Neurological Surgery, University of Wisconsin-Madison, Madison, WI USA

**Keywords:** Balloon catheter, Stent, Stroke, Blood flow

## Abstract

**Background:**

Emergency carotid artery stenting (CAS) is a frequent endovascular procedure, especially in combination with intracranial thrombectomy. Balloon guide catheters are frequently used in these procedures. Our aim was to determine if mechanical aspiration through the working lumen of a balloon occlusion catheter during the steps of a carotid stenting procedure achieve flow rates that may lead to internal carotid artery (ICA) flow reversal which consecutively may prevent distal embolism.

**Methods:**

Aspiration experiments were conducted using a commercially available aspiration pump. Aspiration flow rates/min with 6 different types of carotid stents inserted into a balloon guide catheter were measured. Measurements were repeated three times with increasing pressure in the phantom. To determine if the achieved aspiration flow rates were similar to physiologic values, flow rates in the ICA and external carotid artery (ECA) in 10 healthy volunteers were measured using 4D-flow MRI.

**Results:**

Aspiration flow rates ranged from 25 to 82 mL/min depending on the stent model. The pressure in the phantom had a significant influence on the aspiration volume. Mean blood flow volumes in volunteers were 210 mL/min in the ICA and 101 mL/min in the ECA.

**Conclusions:**

Based on the results of this study, flow reversal in the ICA during common carotid artery occlusion is most likely achieved with the smallest diameter stent sheath and the stent model with the shortest outer stent sheath maximum diameter. This implies that embolic protection during emergency CAS through aspiration is most effective with these models.

## Introduction

The higher rate of embolization and stroke during and immediately after carotid artery stenting (CAS) compared to endarterectomy (Bonati et al. 2015; Economopoulos et al. 2011) led to the development of proximal and distal cerebral protection devices (Knur 2014). Although no randomized controlled study exists showing a benefit of embolic protection devices over unprotected carotid artery stenting, protection is increasingly used and was mandatory in two recent randomized trials (Brott et al. 2016; Rosenfield et al. 2016). In contrast, emergent CAS during thrombectomy procedures is generally performed without protection (Cohen et al. 2015; Lescher et al. 2015). As many as 15–20% of intracranial large vessel occlusions in the anterior circulation are associated with a carotid artery stenosis. Whether emergency CAS in combination with thrombectomy will benefit from the use of protection during the procedure is not yet established.

A common criticism of protection devices is their high cost and more importantly the added procedural complexity associate with their use. This has been reported to correlate with the incidence of periprocedural complications (Barbato et al. 2008; Macdonald et al. 2010). In light of this, a simple modification of established proximal protection has been reported recently (Bhogal et al. 2016). This technique adds no additional cost and can readily be applied for CAS during thrombectomy procedures when balloon guide catheters and an aspiration pump are used. The method utilizes a balloon guide catheter to achieve flow arrest in the common carotid artery (CCA) with continuous aspiration during stent deployment. However, it is unclear how effectively complete flow arrest or even flow reversal in the internal carotid artery (ICA) can be achieved with this method during all steps of the procedure.

The purpose of the present study was to evaluate achievable aspiration flow rates through a large balloon guide catheter at different stages of deployment with five different stents. The aspiration flow rates were compared to the internal and external carotid flow rates measured in healthy volunteers using 4D flow MRI.

## Materials and methods

### Phantom

**Fig. 1:**
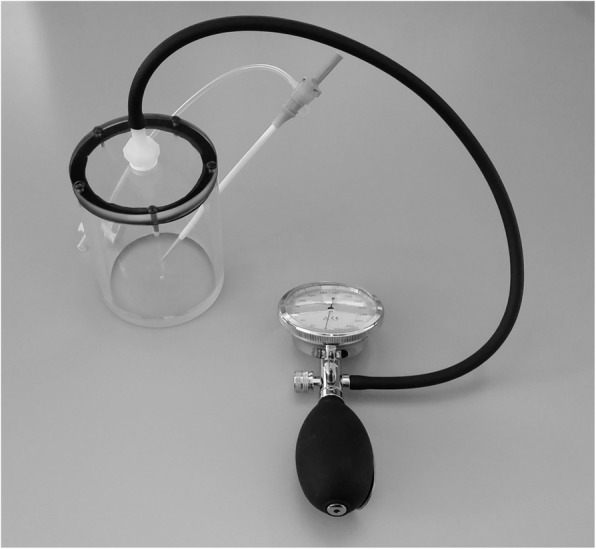
Structure of the fluid-container phantom. The air-tight container allowed aspiration under different pressures to mimic different common cartid artery-stump-pressures. The pressure in the container was controlled via a sphyngomanometer and was adjusted manually during aspiration

A custom made, airtight 800 mL Plexiglas container with a built-in 10 french (10F) sheath (Terumo, Tokyo, Japan) attached to a sphyngomanometer was used for all measurements (Fig. 1).

### Aspiration experiments

For each experiment, the total volume of fluid aspirated over 1 min was measured.

To mimic the viscosity of blood, a 60:40 mixture (by volume) of water and glycerol was selected for use in the trial for all aspiration experiments (Summers et al. 2005). Experiments were repeated three times with the pressure in the phantom (transmural pressure) set manually to 50 mmHg, 75 mmHg and 100 mmHg in order to mimic the effect of different stump pressures.

A 9F balloon guide catheter with an inner lumen diameter of 0.085 in / 2.1 mm and a length of 95 cm (9 F Merci, Concentric Medical/Stryker Neurovascular, Fremont, CA, USA) connected to a commercially available aspiration pump (Penumbra MAX Aspiration Pump, Penumbra, Alameda, CA, USA) was used for all aspiration experiments. The pump achieved a maximum pressure (differential to atmospheric pressure) of − 26 inHg (− 660 mmHg). Before each test run, the catheter and the tubing leading to the pump were filled with blood mimicking fluid so that no air remained in the system. A valve was shut until the maximum differential pressure had been reached and was opened subsequently. Volumetric flow rates of the aspirated fluid using six different FDA approved carotid artery stent models, introduced through the working lumen of the balloon guide catheter, were evaluated (technical details are summarized in Table 1).

**Table 1:** Overview of the dimensions of the size of the deployment system: Sheath diameter refers to the maximum diameter of the stent deployment sheath, sheath length refers to the length of the maximum diameter, which is frequently limited to the most distal part where the stent is located in the deployment system, shaft diameter refers to the diameter of the proximal part of the deployment system, stent diameter refers to the maximum stent diameter possible with the mentioned deployment system size, stent length was kept 30 mm for all systems. Outer sheath and shaft dimensions of the evaluated stent models

	Sheath diameter	Sheath length	Shaft diameter	Stent diameter^a^	Stent length
Precise 7 mm	5F (1.65 mm)	215 mm	2.9F (1 mm)	7 mm	30 mm
Precise 10 mm	6F (1.98 mm)	215 mm	2.9F (1 mm)	10 mm	30 mm
Acculink	6F (1.98 mm)	50 mm	4.4F (1.5 mm)	10 mm	30 mm
Xact	5.7F (1.9 mm)	240 mm	NA	8 mm	30 mm
Protégé	6F (1.98 mm)	270 mm	NA	8 mm	30 mm
Casper	5.2F (1.73 mm)	NA	NA	All sizes	All sizes

^a^Sheath diameter does not vary with stent diameter for Acculink, Xact, Protégé

Precise 10/30 mm (Cordis, Milpitas, CA, USA), distal outer sheath diameter 1.98 mm (6F), length 215 mm, 2) Precise 7/30 mm (Cordis, Milpitas, CA, USA) distal outer sheath diameter 1.65 mm (5F), length 215 mm, 3) Acculink RX 10/30 mm (Abbott vascular, Santa Clara, CA, USA) distal outer sheath parameter 1.98 mm (6F), length 50 mm, 4) Xact 8/30 mm (Abbott vascular, Santa Clara, CA, USA) distal outer sheath parameter 1.9 mm (5.7F), length 240 mm, and 5), Protégé RX 8/30 mm (ev3/Medtronic, Minneapolis, MN, USA) outer sheath parameter 1.98 mm (6F), length 270 mm, 6) Casper 6/30 mm (Microvention, Aliso Viejo, CA, USA).

Aspiration experiments were sequenced to mimic the steps of a carotid stenting procedure: 1) Stent delivery catheter in guidecatheter with tip of distal outer sheath at the distal end of the guidecatheter, 2) distal outer sheath of stent delivery catheter after stent deployment at the distal end of the guidecatheter. An additional measurement was undertaken with the distal outer sheath fully advanced through guide catheter with catheter shaft remaining in guide catheter, however this is unlikely during CAS due to the length of the deployment system of the majority of the stents.

After 1-min aspiration, the valve to stop aspiration was closed and the content of the pump bucket was transferred into a measurement cylinder to determine the amount of fluid aspirated. With this setting, the catheter did not have to be primed during injections with the same stent system inserted. After each change of stent system, the catheter and tubing were primed with blood mimicking fluid anew.

As a final step, both water and the 60:40 water-glycerol mixture were aspirated though the empty guide catheter over 1 min each.

The influence of distal outer stent sheath length and distal outer stent sheath diameter on flow rates were evaluated based on three of the evaluated carotid stent models. Two out of five stent models (Acculink RX 10 mm and Precise 10 mm) had the same distal outer stent sheath diameter but a different stent sheath length.

Two stent models (Precise 7 mm and Precise 10 mm) had the same distal outer stent sheath length but a different stent sheath diameter.

The distal outer stent sheath describes the most distal part with the largest diameter of the stent catheter where the stent is mounted. The diameter of this part of the stent catheter primarily determines the aspiration flow rates when inserted in a guide catheter. To calculate the axial (cross-sectional) surface area of the guide catheter lumen as well as axial surface area of stent sheaths and shafts, we utilized the equation (Eq. 1):

1 $$ A=\pi {r}^2 $$

Where r refers to the radius (diameter/2) of the inner lumen of the guide catheter as well as the diameter /2 of stent sheaths and shafts.

To calculate the remaining axial surface of the guide catheter with the different devices inserted, we utilized the following equation (Eq. 2):

2 $$ {A}_{guide}-{A}_{device} $$

Where *A*_*guide*_ refers to the axial surface area of the inner lumen of the guide catheter and *A*_*device*_ refers to the axial surface area of the device.

Poiseuille’s law solved for the flow rate defines the parameters that determine the flow rate through a tube (Eq. 3):

3 $$ Q=\frac{{\Delta  P\pi r}^4}{8\mu L} $$

Where *∆P* refers to the pressure difference, *μ* to dynamic viscosity of the fluid, *L* to the length of the tube, *Q* to the volumetric flow rate and *r* to the radius of the tube (Munson 2013).

The equation illustrates that the dynamic viscosity, pressure difference and length of the tube affect volumetric flow rate linearly whereas the tube radius affects flow rate to the fourth power. However, Poiseuille’s law assumes laminar flow.

In order to evaluate the effects of distal outer sheath length and diameter, we compared the difference in sheath length in percent to the difference as well as the difference in sheath diameter in percent to the change in flow rate in percent.

To determine the effect of pressure inside the phantom, paired 2-sided t-test were conducted to test for significant differences in aspiration volumes between measurements at 50 mmHg and 75 mmHg, between 50 mmHg and 100 mmHg and between 75 mmHg and 100 mmHg. A *p*-value < 0.05 was deemed significant).

### 4D-flow MRI measurements

This prospective analysis was Health Insurance Portability and Accountability Act (HIPAA) compliant and approved by the local institutional review board (IRB). Ten volunteers with no known significant health problems were recruited. All volunteers signed an IRB-approved informed consent form. Data were acquired on a 3 T MR imaging system (MR750, GE Healthcare, Waukesha, WI) with an 8-channel head and neck coil (neurovascular coil, GE healthcare). Volumetric, cardiac time-resolved PC MRI data with three-directional velocity encoding were acquired with a 3D radially undersampled sequence (PC VIPR), with the following imaging parameters: velocity encoding (VENC): 150 cm/s, imaging volume. (22x22x16 cm^3^), acquired isotropic spatial resolution 0.7 mm^3^, repetition time (TR)/echo time (TE): 7.4/2.7 ms, 14,000 projection angles, flip angle 10°, bandwidth 83.3 kHz (Gu et al. 2005). Time averaged magnitude and velocity data were generated via an offline reconstruction for all subjects. For each subject, cardiac triggers were collected from a photoplethysmogram on a pulse oxymeter worn on the subject’s finger during the exam. Blood pressure was recorded for all volunteers. In the acquired 3D time resolved datasets, measurement planes were placed at the origin of the external and internal carotid artery. Volumetric blood flow (mL/min) was calculated for each vessel.

## Results

### Aspiration experiments

**Fig. 2:**
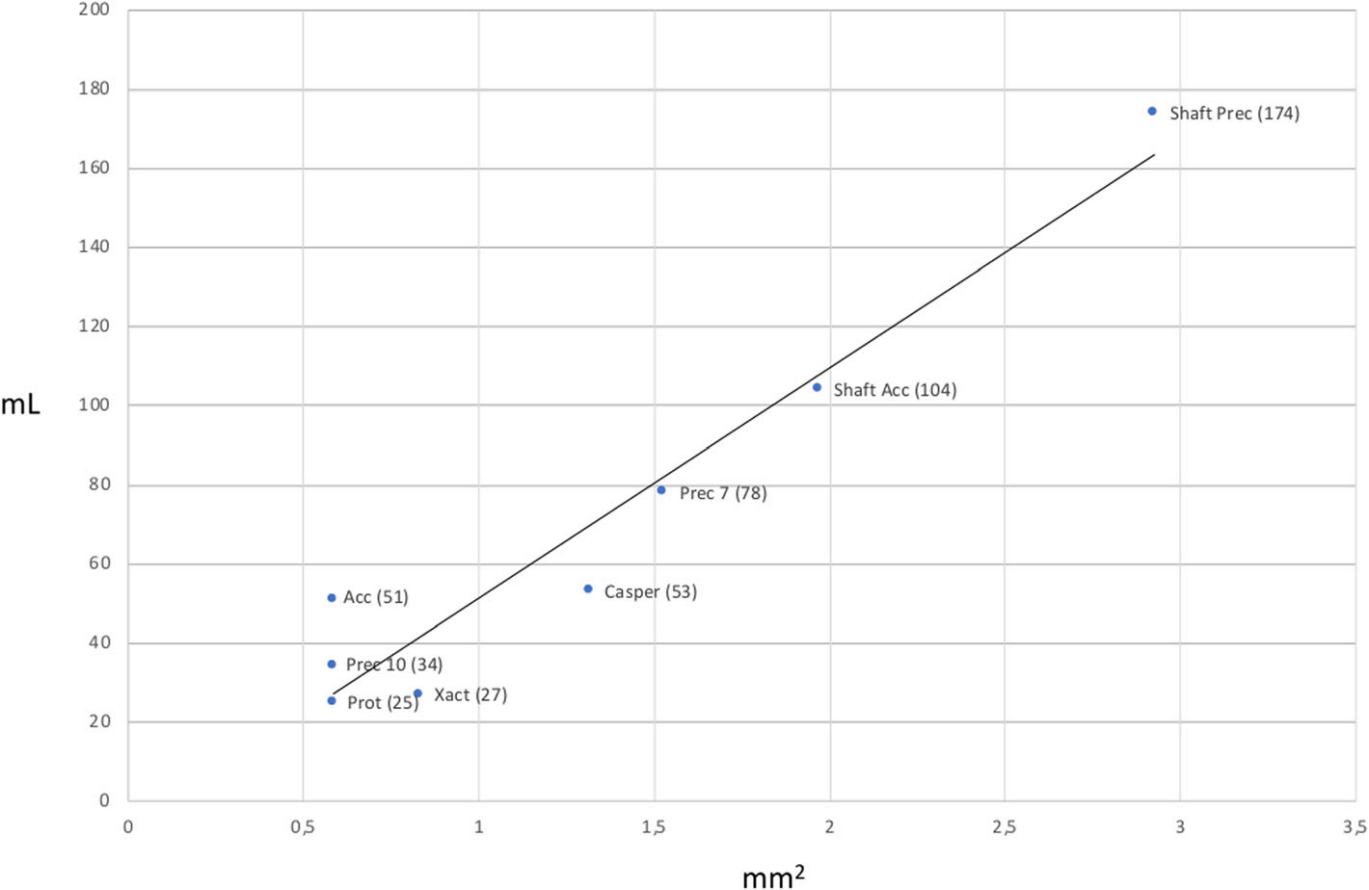
Aspiration volume flow rates at a pressure of 50 mmHg (y-axis, mL = Milliliters) plotted vs remaining axial surface area of the guide catheter working lumen after the stent catheter was introduced (x-axis, mm^2^ = Millimeters square). Plotted are aspiration rates with all undeployed stents and the stent catheter shafts of the models where size information was available within the working lumen of the guide catheter (Abbreviations: Acc: Acculink, Prec 7/10: Precise 7 mm/10 mm, Prot: Protégé. The numbers in brackets show the volumetric aspiration rates). The graph depicts a nearly linear relation of flow rates and luminal surface area, which shows that laminar flow is not present

Aspiration flow rates with the different stents inserted in the guide catheter are summarized in Table 2 and graphically depicted in Fig. 2.

**Table 2:** Overview over the aspiration flow rates in Millilters / minute (mL/min) with the different devices inserted in the guide catheter at 50, 75 and 100 mmHg pressure within the phantom. The shaft values apply once the outer stent sheath is completely pushed out of the distal end of the balloon catheter and only the shaft remains within the catheter

	50 mmHg	75 mmHg	100 mmHg
Precise 7 mm	78	82	82
Casper	53	56	56
Acculink	51	50	53
Precise 10 mm	34	35	40
Xact	27	30	32
Protégé	25	25	25
Precise shaft	174	179	181
Acculink shaft	90	104	100
Xact shaft	99	110	124
Protégé shaft	61	67	71

The largest volumetric flow rate was achieved with the 7 mm Precise stent (78 mL at 50 mmHg / 82 mL at 75 mmHg / 82 mL at 100 mmHg) followed by the 6 mm Casper stent (53 mL/56 mL/56 mL), 10 mm Acculink stent (51 mL/50 mL/53 mL), 10 mm Precise stent (34 mL/35 mL/40 mL), 8 mm Xact stent (27 mL/30 mL/32 mL) and the 8 mm Protégé stent (25 mL/25 mL/25 mL).

After deployment of the stents, the following flow rates were measured: Precise 7 mm: 82/88/92 mL/min, Casper 6 mm: 52/58/59), Acculink 10 mm: 50/49/54 mL, Precise 10 mm: 39/44/49 mL, Xact 8 mm: 31/28/31 mL, Protégé 8 mm: 23/25/24 mL.

With the outer sheath of the stent delivery catheter fully advanced through the catheter and only the stent delivery catheter shaft remaining in the guide catheter, the following flow rates were recorded: Precise 10 mm/7 mm (2.9F): 174/179/181 mL/min, Acculink (4.4F): 90/104/100 mL/min, Xact (no size information): 99/110/124 mL/min, Protégé (no size information): 61/67/71 mL/min. Xact and Protégé systems had to be introduced in the catheter until mechanical stop to achieve this position.

Mean aspiration volumes of all performed measurements were 71 mL at 50 mmHg phantom pressure, 75.8 mL at 75 mmHg and 78.7 mL at 100 mmHg.

Paired, two-sided t-tests revealed a significant difference between aspiration volumes at 50 mmHg and 75 mmHg (***p*** **= 0.013**) and between 50 mmHg and 100 mmHg (***p*** **= 0.01**) but not between 75 mmHg and 100 mmHg (***p*** **= 0.12**).

Aspiration of water at room temperature and atmospheric pressure through the balloon guide catheter resulted in a volumetric flow rate of 383 mL/min. Aspiration of the 60:40 water:glycerol mix resulted in a volumetric flow rate of 237 mL/min.

### 4D-flow MRI measurements

Volumetric blood flow rates were successfully measured in all 10 volunteers, providing 20 data points for ICA and 20 for external carotid artery (ECA) measurements. The measurements in the external carotid arteries revealed a mean volumetric flow rate of 100.9 mL/min (range: 44.8–193.3, standard deviation (SD): 34.5). Measurements in the internal carotid arteries revealed a mean volumetric flow rate of 210.4 mL/min (range: 124.3–334.4, SD: 60.6). These values are in good agreement to values measured with 2D PC MRI in a comparable cohort (Oktar et al. 2006) Blood pressure measurements revealed a mean arterial pressure of 90.3 mmHg (SD: 8.2 mmHg).

## Discussion

We demonstrate that, depending on the stent used, mechanical aspiration through the lumen of a balloon occlusion catheter during the various steps of carotid stent delivery and deployment allow minimum flow rates of 82–25% of unrestricted antegrade ECA flow or 12%–39% of total unrestricted antegrade ICA flow. The flow volume values are based on blood flow rates measured in 10 healthy volunteers.

Our results show that the subset of patients where a full flow reversal might be achieved with aspiration during CCA balloon occlusion will decrease as the diameter and length of the outer stent sheath increases. Furthermore, given the theoretical consideration that a full flow reversal in the ECA is highly unlikely due to the change in blood pressure distal to the occlusion site, the highest achievable aspiration flow volumes are likely to overcome reversed ECA flow in a subset of patients and therefore lead to a flow reversal distal to the CCA occlusion. Flow reversal during emergency CAS, especially after recanalization of acute carotid occlusion with potentially large clot burden, may decrease distal embolism. Flow reversal in the ICA is most likely to be achieved when the (up to) 8 mm diameter Precise stent is used. Even though not included in the experiment, the (up to) 8 mm diameter Carotid Wallstent is very likely to exhibit similar aspiration flow volumes compared to the Precise stent due to its identical diameter of the outer stent sheath. Second highest flow rates were achieved with the Casper stent, a double layer micromesh stent that utilizes a 5.2F delivery system for all stent diameters. The Acculink stent with its short distal outer sheath achieves similar high aspiration rates compared to Casper and also utilizes one deployment system size for all stent diameters. We excluded potential differences from stent length by choosing the same length for all models (30 mm).

In this study, the highest aspiration volumes excel 80% of antegrade ECA flow. This would imply that if ECA flow under CCA occlusion is fully reversed, more than 80% of reversed ECA flow volume could be aspirated. The scenario of full flow reversal, however, is unlikely as the arterial pressure decreases strongly distal to the occlusion site. In one small case series, Ohki et al. (2001) measured ICA and ECA stump pressures during carotid endarterectomy and found a mean arterial pressure of 62.2 mmHg in the ICA and of 52.8 mmHg in the ECA resulting in a mean gradient of 10.4 mmHg between ECA and ICA. The pressure gradient from ECA to ICA drives antegrade flow in the ICA. However, the resulting flow volume from ECA to ICA in case of CCA occlusion cannot be easily estimated and is dependent of the collaterization of the ECA and ICA. Furthermore, these factors are highly variable among individuals.

To address the uncertainty of individual blood flow characteristics in the ECA and ICA under balloon occlusion, DSA may be performed through the guide catheter under balloon occlusion. Using this method, a qualitative analysis of flow directions in the ECA and its branches as well as the ICA may be achieved. Together with the assessment of the intracranial collateralization (Henderson et al. 2000), conclusions about the efficacy of the aspiration during CAS might thus be possible. Semi quantitative methods for hemodynamic evaluation might be beneficial in this context (Strother et al. 2010).

Our study showed significantly higher aspiration volumes with higher pressure in the phantom mimicking higher CCA stump pressure. However, higher carotid stump pressure means efficient collateralization during CCA occlusion. This might counterbalance the higher aspiration volumes, especially when collateralization is directed from ECA to ICA.

The stents evaluated in this study were the most commonly used carotid artery stents in the US (Giri et al. 2014). The most popular stent in Europe, the Carotid Wallstent, was not evaluated. However, the outer stent sheath diameters of this stent model are identical to those of the Precise system with different outer sheath sizes dependent on stent size (1.67 mm up to 8 mm stent diameter, 1.97 mm for 9/10 mm stent diameter). The experimental setup used is easily applicable as a proximal protection technique for emergent stenting procedures during thrombectomy for ischemic stroke (Cohen et al. 2015; Lescher et al. 2015). It could also be used for emergent carotid stenting procedures with intraluminal thrombus (Imai et al. 2007).

With regard to the evaluation of aspiration rates through catheters, our study shows the importance of using fluid with blood mimicking properties; this is not generally performed (Hu and Stiefel 2016; Simon and Grey 2014). Even with large catheters, the differences in aspiration rates compared to water are still very large (roughly 60% in our study).

Finally, the results of the aspiration experiments show that, under the conditions of our experiments, flow of the aspirated fluid is not laminar but turbulent. In the condition of laminar flow, remaining axial catheter surface would affect flow rates by the fourth power (Eq. 3). In contrast, aspiration rates follow a more linear relation to the remaining axial catheter surface (Fig. 2).

This study has several limitations. We measured blood flow rates in healthy volunteers; these might differ from patients with carotid arteriosclerotic disease. However, as flow rates to the brain decrease with age (Yang et al. 2016), actual flow rates during CCA occlusion in patients might be rather lower than higher than the ones measured in this study.

## Conclusion

In this study, the highest aspiration flow rate through a 9F balloon occlusion catheter was 3.3-fold higher than the lowest depending on the introduced carotid artery stent model. The phantom experiments indicate that the efficacy of distal embolism protection through aspiration during CCA occlusion increases strongly if stents with a low outer stent sheath profile or short maximum stent sheath diameter are used. Furthermore, this study highlights the potential importance of using large balloon occlusion catheters as guiding catheters for emergency CAS procedures during thrombectomy for ischemic stroke (Velasco et al. 2016).

## Data Availability

All data generated or analysed during this study are included in this published article.

## Abbreviations

ICAf: Internal carotid artery
ECA: External carotid artery
CCA: Common carotid artery
CAS: Carotid artery stenting
